# The rationale and design of TransCon Growth Hormone for the treatment of growth hormone deficiency

**DOI:** 10.1530/EC-17-0203

**Published:** 2017-09-25

**Authors:** Kennett Sprogøe, Eva Mortensen, David B Karpf, Jonathan A Leff

**Affiliations:** 1 Ascendis Pharma A/SHellerup, Denmark; 2 Ascendis PharmaInc., Palo Alto, California, USA

**Keywords:** long-acting growth hormone, TransCon GH

## Abstract

The fundamental challenge of developing a long-acting growth hormone (LAGH) is to create a more convenient growth hormone (GH) dosing profile while retaining the excellent safety, efficacy and tolerability of daily GH. With GH receptors on virtually all cells, replacement therapy should achieve the same tissue distribution and effects of daily (and endogenous) GH while maintaining levels of GH and resulting IGF-1 within the physiologic range. To date, only two LAGHs have gained the approval of either the Food and Drug Administration (FDA) or the European Medicines Agency (EMA); both released unmodified GH, thus presumably replicating distribution and pharmacological actions of daily GH. Other technologies have been applied to create LAGHs, including modifying GH (for example, protein enlargement or albumin binding) such that the resulting analogues possess a longer half-life. Based on these approaches, nearly 20 LAGHs have reached various stages of clinical development. Although most have failed, lessons learned have guided the development of a novel LAGH. TransCon GH is a LAGH prodrug in which GH is transiently bound to an inert methoxy polyethylene glycol (mPEG) carrier. It was designed to achieve the same safety, efficacy and tolerability as daily GH but with more convenient weekly dosing. In phase 2 trials of children and adults with growth hormone deficiency (GHD), similar safety, efficacy and tolerability to daily GH was shown as well as GH and IGF-1 levels within the physiologic range. These promising results support further development of TransCon GH.

## Introduction

Following secretion from the anterior pituitary, human growth hormone (GH) is transported throughout the body, exerting its effects via GH receptors on virtually every cell of the body. In addition to muscle and bone growth, GH is critical for a wide variety of bodily functions, including adiposity regulation, glucose control, lipid metabolism, and cognition (1, 2, 3). These effects are mediated both directly by GH and indirectly by insulin-like growth factor (IGF-1). In target organs (e.g., liver) and peripheral tissue (e.g., growth plate), GH induces production of IGF-1. The liver is the primary source of circulating IGF-1, which regulates endogenous GH production via a negative feedback loop, while tissue-derived IGF-1 acts in both an autocrine and paracrine fashion (4). GH and IGF-1 thus work in concert, with IGF-1 augmenting the anabolic actions of GH while opposing the hyperglycemic and lipolytic effects of GH (5).

Growth hormone deficiency (GHD), which may be congenital or acquired, can afflict any age and manifests itself similarly across age ranges. Children with GHD suffer from short stature, increased and disproportionately distributed body fat, lipid abnormalities and decreased bone mineral density while adults with GHD suffer similar consequences except short stature (6, 7).

Because of the ubiquity of GH receptors, it is important that a GHD therapeutic be small enough to permeate all tissues and carry out all the beneficial effects of GH. GHD is treated with replacement therapy, i.e., recombinant daily human GH (somatropin, molecular weight 22 kDa). Demonstrated to be safe and effective, somatropin is administered subcutaneously (SC) on a daily basis. This is particularly burdensome given that patients with GHD usually require ongoing treatment for many years resulting in thousands of injections. Understandably, many patients are, or eventually become, noncompliant, leading to suboptimal outcomes (8, 9, 10, 11, 12, 13).

Studies of GH infusion have demonstrated that continuous exposure to unmodified GH is a safe and effective alternative to daily injections (14, 15, 16). Thus, to ameliorate injection fatigue and improve outcomes, development of long-acting formulations has garnered considerable interest. As early as 1979, Lippe and coworkers evaluated the efficacy of a depot GH preparation in GHD children (17). Since then, multiple companies have attempted to develop a long-acting growth hormone (LAGH).

In 2015, the Growth Hormone Research Society convened a workshop to discuss LAGH development. The group theorized that by decreasing injection frequency, LAGH would improve adherence and therefore outcomes. A number of potential safety issues related to LAGH were identified, including (i) supraphysiological elevations of GH and/or IGF-1, (ii) trough GH concentrations above normal physiological levels, (iii) fluctuating IGF-1 levels, (iv) elevated IGF-1 in the absence of GH bioactivity, (v) nonphysiological tissue distribution due to distinct biological features of the products and (vi) the specific chemical composition of each LAGH product (11).

To date, nearly 20 LAGHs have reached various stages of development (Table 1). Most have failed, some were successfully launched but failed to gain commercial success, two are currently only available in narrow geographic regions, while the remaining are in various stages of clinical development (11). With the goal of satisfying an unmet need, work to develop a safe and effective LAGH continues.

**Table 1:** A summary of long-acting growth hormones categorized by development approach.

**Approach**	**Company**	**Product**	**Pediatric GHD development status**
Unmodified GH – Half-life extension achieved by the slow-release of somatropin from polymeric depot, crystal, or prodrug	Genentech, Inc.	Nutropin Depot GH encapsulated in polylactide-coglycolic acid microparticles	Approved in the U.S; later withdrawn
	LG Life Sciences, Ltd.	LB03002 GH encapsulated in sodium hyaluronate microparticles	Approved but not marketed in Europe; available in South Korea
	Altus Pharmaceuticals, Inc.	ALTU-238 GH crystallization	Discontinued
	Ascendis Pharma A/S	TransCon GH Transiently PEGylated GH prodrug	Phase 3
Modified GH – Half-life achieved by increasing molecular size (except NNCO195-0092, which is modified with a small albumin affinity tag)
	GeneScience Pharmaceuticals Co., Ltd.	Jintrolong Permanently PEGylated GH	Available in China
	Pfizer, Inc.	PHA-794428 Permanently PEGylated GH	Discontinued
	Novo Nordisk A/S	NNCl126-0083 Permanently PEGylated GH	Discontinued
	Ambrx, Inc.	ARX201 Permanently PEGylated and mutated GH	Discontinued
	Teva Pharmaceutical Industries, Ltd.	TV-1106 GH fused to albumin	Discontinued
	Versartis, Inc.	VRS-317 GH fused to half-life extension technology polypeptides	Phase 3
	OPKO Health, Inc.	MOD-4023 GH fused to carboxyterminal peptides	Phase 3
	Novo Nordisk A/S	NNCO195-0092 Mutated GH attached to an albumin affinity tag	Phase 2
	Genexine, Inc., and Handok, Inc.	GX-H9 GH fused to an Fc fragment	Phase 2
	Hanmi Pharmaceutical Co., Ltd.	LAPS-rhGH/HM10560A GH fused to an Fc fragment	Phase 2

## True replacement therapy with convenient dosing

The fundamental challenge of developing a LAGH is to create a more convenient GH dosing profile while retaining the excellent safety, efficacy and tolerability profile of daily GH. With GH receptors in essentially all tissues, replacement therapy should achieve the same tissue distribution and effects of endogenous (and daily) GH while maintaining levels of GH and resulting IGF-1 within the physiologic range.

To create a LAGH that extends the GH half-life thereby allowing less frequent dosing, two basic approaches have been followed: (a) combine unmodified GH with a prolongation technology (a depot, crystal or prodrug) or (b) modify GH in such a way (protein enlargement or albumin binding) that the GH analogue has a longer half-life.

### Formulations based on unmodified GH

Only LAGHs based on unmodified GH have ever gained approval in Europe or the United States. Nutropin Depot is the only LAGH ever approved by the Food and Drug Administration (FDA). It consists of recombinant human GH encapsulated in biodegradable polylactide-coglycolic acid (PLGA) polymer microspheres. Unmodified GH was released slowly from the microsphere into SC tissue (18). Nutropin Depot only achieved an annualized height velocity (HV) of 8.2 cm/year that, according to a letter issued by the FDA, was not comparable to daily GH (19). The low growth rates observed with Nutropin Depot may have been related to lower GH exposure (due to suboptimal pharmacokinetics; PK) compared to daily injections using standard doses of 0.30 mg/kg/wk (20, 21). In addition to subpar efficacy, the Nutropin Depot formulation also required multiple injections in patients weighing more than 26 kg. Large injection volumes along with injection site reactions rendered the product poorly tolerated (18, 20), and it was eventually withdrawn from the market (11, 13).

LB03002 consists of unmodified GH embedded in sodium hyaluronate microparticles suspended in triglycerides and provided equivalent efficacy to that of daily GH (Genotropin) both in terms of annualized HV in children as well as decreased fat mass in adults (22, 23, 24). However, in children receiving LB03002, non-serious injection site reactions and non-neutralizing anti-GH antibodies were observed, the latter with an incidence 5 times higher than daily GH, although without impact on safety. Neutralizing antibodies were not detected except in one patient with a GH gene deletion (25). In its assessment report, the European Medicines Agency (EMA) highlighted that GH (and resulting IGF-1) should not exceed physiological levels when used as substitution therapy based on the concern that anabolic compounds may promote tumor proliferation (25). It concluded that GH and IGF-1 exposure induced by LB03002 were similar to daily GH. This product was approved by the EMA but not marketed in the European Union; it is available in South Korea.

### Formulations based on modified GH

Protein enlargement technologies have been used to prolong the half-life of GH by permanently increasing molecular size. This can be achieved by attaching one or more synthetic peptides or other polymers such as polyethylene glycol (PEG) or by fusion of GH with other proteins such as albumin, non-naturally occurring repeat amino acid sequences (e.g., XTEN), carboxyterminal peptide (CTP) or Fc antibody fragments. These increases in molecular size reduce renal filtration and interactions with the GH receptor (reducing receptor-mediated degradation), thus prolonging half-life.

Permanent PEGylation, the covalent binding of one or more PEG molecules to an active drug, is a technology frequently used to extend the half-life and improve other pharmacological properties of proteins and peptides. At least a dozen PEGylated biopharmaceuticals have been approved in the United States and Europe (26). In addition, PEG has found widespread use in the industrial, cosmetic and food industries.

Creating a LAGH by permanent PEGylation has been attempted repeatedly and led to one approval in China (11). Other programs have failed at various stages of clinical development. Altered pharmacology of the protein-enlarged GH has led to unexpected outcomes, for example, injection site lipoatrophy in patients administered permanently PEGylated GH, PHA-794428; a higher incidence was observed among females, and the incidence increased with repeat injections (27), suggesting a change in adipocyte susceptibility to the lipolytic activity of the protein-enlarged GH over time. The findings of lipoatrophy led Pfizer, Inc., to discontinue development of PHA-794428 (28).

Novo Nordisk A/S developed another PEGylated LAGH, NNC126-0083, which showed potentially acceptable PK and pharmacodynamic (PD) profiles in adults with GHD following once-weekly administration (29). However, a satisfactory IGF-1 profile was not achieved in children with GHD (30), and as a result, development was discontinued (31, 32).

While PEG remains the best characterized inert polymer for modifying biopharmaceuticals and has demonstrated safety and tolerability in chronic settings, repeat parenteral administration of PEGylated proteins to animals has in some cases been associated with vacuolation – the formation of enclosed intracellular compartments for clearance of debris – in macrophages and/or histiocytes of various organs, including renal tubular cells (33). Formation of vacuoles in the choroid plexus of cynomolgus monkeys have been reported for a permanently PEGylated GH analogue, ARX201, which was being developed by Ambrx (34). Vacuolation, which occurs mainly in phagocytes, has not been linked to organ dysfunction in toxicology studies, and the body of evidence for approved PEGylated biopharmaceuticals has not identified any clinical consequences for compounds where vacuolation was observed in toxicology studies (35).

Active transport (movement of molecules across cell membranes) by receptors may be a determining factor as to which drugs cause vacuolation and which do not. Based on repeat-dose toxicity studies of a permanently PEGylated GH formulation administered SC to cynomolgus monkeys, vacuolation has been observed within cells with GH receptors, specifically choroid plexus ependymal cells. When the unconjugated PEG molecule itself (i.e., free PEG without GH attached) was administered, a 10-fold higher dose was required to detect it within ependymal cells. In other words, permanently PEGylated GH is taken up into ependymal cells much more readily than unconjugated PEG. This is likely a result of receptor-mediated transport of GH in which PEG uptake is an incidental consequence compared to nonspecific cellular uptake (pinocytosis) of PEG alone (36).

A review of published data and information provided by a survey conducted by BioSafe (a committee within BIO, the Biotechnology Industry Organization) stated that the body of evidence available for approved PEGylated biopharmaceuticals has not identified any clinically reported functional consequences for compounds where vacuolation was observed in toxicology studies (35).

TV-1106 is a long-acting GH developed by Teva Pharmaceutical Industries, Ltd. TV-1106 comprises human serum albumin (HSA) genetically fused to the N-terminus of GH. Despite both GH and albumin being naturally occurring, the fusion protein had a high immunogenic potential leading to neutralizing antibodies in 4 of 48 (8.3%) TV-1106-treated subjects in a phase 2 trial of children with GHD. When Teva Pharmaceutical Industries, Ltd., reassessed the benefit/risk balance of TV-1106 and the likelihood of regulatory success, the trial and product development were terminated (37).

Somavaratan (VRS-317) is a fusion protein with a molecular weight of 119 kDa being developed by Versartis, Inc., for twice monthly injections. The 22 kDa GH domain is enlarged by two chains of hydrophilic, non-naturally occurring amino acid sequences (XTEN) added to the N- and C-termini of GH. This enlargement prolongs the half-life of GH by increasing its hydrodynamic size and decreasing receptor-mediated clearance through a reduction in receptor binding (38). Somavaratan is currently in phase 3 clinical development. Adverse events following somavaratan administration were reported to be similar to daily GH in a phase 2 trial of children with GHD. However, the trial did not include an active comparator (38). Depending on the somavaratan dose, annualized HV was reported to be 7.58–8.61 cm/year (38). However, neutralizing antibodies have been reported for 2 of 64 (3%) subjects administered somavaratan in the phase 2 trial of children with GHD (39, 40). The incidence of non-neutralizing anti-GH antibodies has not been reported.

Developed by OPKO Health Inc., MOD-4023 is a once-weekly administered GH fusion protein consisting of three carboxyterminal peptide (CTP) copies of the beta chain of human chorionic gonadotropin. MOD-4023 has demonstrated growth similar to daily GH in a phase 2 trial of children with GHD with a phase 3 trial recently initiated (41). However, a recent phase 3 trial in adults with GHD missed its primary endpoint of truncal fat mass reduction; with only a 0.4 kg weight decrease in the active arm, a statistical difference from placebo was not observed (42, 43).

A different human GH derivative was formulated by Novo Nordisk A/S in which a side chain with a terminal fatty acid (with noncovalent albumin-binding properties) is attached to a single point mutation in the GH backbone, thus decreasing clearance and increasing half-life (44). Somapacitan (NNbib195-0092) binds tightly, but reversibly, to human albumin, leaving an estimated 0.04% of somapacitan unbound (45). Phase 3 data in adults with GHD have demonstrated that once-weekly somapacitan was more convenient than daily GH (46), supporting the idea that LAGH may improve compliance. Somapacitan is currently in phase 3 development for adults with GHD and in phase 2 development for pediatric GHD.

Co-developed by Handok, Inc., and Genexine, Inc., GX-H9 is a recombinant human GH in which GH is fused to an Fc antibody fragment (hybrid Fc) to extend half-life. A phase 2 trial in children with GHD is ongoing (47).

LAGH development based on GH modification by molecular enlargement and albumin binding creates new active GH analogues. These analogues are designed to support dosing frequencies from 1 to 2 weeks and up to 1 month. As such, upon injection, these GH analogues are absorbed into the bloodstream, leading to supraphysiological GH activity (at maximum analogue concentrations shortly after administration) greatly exceeding that obtained with daily GH administration. With time, concentration of the modified GH analogue falls until subsequent dosing, again leading to GH activity levels above the normal range. Supraphysiologic GH may be associated with increased cardiac output, concentric left ventricular remodeling, cardiomyopathy and acromegalic symptoms (48, 49, 50, 51, 52). Clinical manifestations of GH hypersecretion evolve slowly and can take years to diagnose (52), a noteworthy consideration when developing a LAGH. Given the potential for adverse effects secondary to supraphysiologic GH, regulators and others have expressed concerns (11, 25).

Permanently enlarging GH may compromise tissue distribution. To assess the effect of molecular size on tibial growth plate penetration, Farnum and coworkers used a murine model and demonstrated that 40 kDa and larger dextrans do not enter or diffuse through the growth plate (53). Similarly, Gill and coworkers showed an inverse relationship between the molecular size of therapeutic proteins and extravascular tissue distribution in a human whole-body, physiologically based PK model (54). Additionally, in patients with hepatitis C treated with PEGylated interferons, Caliceti and coworkers found that molecular weight affected relapse rates; PEGylated interferons with higher molecular weight (and therefore lower volumes of distribution) led to higher relapse rates due to the lower chance of infiltrating extravascular tissue (55).

Interestingly, from fish to humans, GH is highly conserved across species, ranging from 19.4 to 22 kDa in size (56). This suggests evolutionary constraints on the functional GH molecule and the importance of size in maintaining natural tissue penetrance. Unmodified GH may distribute more fully into peripheral tissues, whereas protein-enlarged GH molecules may have restricted access. Like unmodified GH, however, protein-enlarged GH molecules do readily access hepatic GH receptors via fenestrated (open) hepatic sinusoidal endothelium, stimulating hepatic IGF-1 production. Such imbalances within peripheral tissue vs organ distribution have been described for another protein-enlarged hormone, namely permanently PEGylated insulin, in which increased molecular size favored the liver over peripheral tissue (57).

The GH-IGF system has evolved to provide a distinct advantage to the organism, with IGF-1 augmenting the growth-promoting actions of GH while countering its potentially deleterious effects of hyperglycemia and lipid store depletion (5); the anabolic actions of IGF-1 are synergistic to those of GH, whereas its metabolic actions are antagonistic. When this balance is disrupted, for example, by the administration of certain drugs, the effects of either GH or IGF-1 may predominate, with altered tissue ratios possibly leading to suboptimal therapeutic outcomes.

GH has direct in vivo effects (independent of circulating IGF-1) exerted, in part, through local IGF-1 production (5). As much as 20% of linear growth is estimated to be the result of the direct effects of GH on growing bone (5, 58, 59), a finding described in animal studies and supported by studies of children with GH receptor insensitivity/deficiency. Patients with Laron syndrome treated with IGF-1 demonstrated lower growth rates compared to patients with GHD treated with daily GH, supporting the contribution to growth that both GH and IGF-1 provide (59, 60).

Tissue balance of GH and IGF-1 is important and disruption may lead to undesired effects. For example, a dearth of lipolytic GH in the presence of lipogenic IGF-1 in fat tissue leads to lipogenesis. While chronic administration of GH has been shown to reduce fat mass by approximately 15% (61), unopposed IGF-1 action may lead to accumulation of fat and increases in body mass index (38, 62). Studies of patients with Laron syndrome demonstrate this phenomenon; long-term treatment with recombinant IGF-1 is associated with an increase in adiposity (63).

Lipoatrophy, a potentially disfiguring condition that has caused the discontinuation of at least one LAGH (28), is frequently reported among patients administered enlarged GH proteins (27, 64, 65, 66). By contrast, it is thought that unmodified GH carries a lower risk; lipoatrophy is not considered a problem for daily GH. Although the pathogenesis remains unclear (67), high localized concentrations of lipolytic GH in SC tissue may be a contributing factor. Lipoatrophy seems to be more frequent among females possibly because of SC adipose tissue differences between genders (27), making it critically important that studies evaluating safety and tolerability of LAGHs include women.

## Integrating previous programs – a target product profile

Lessons learned from past development attempts have shown that the optimal LAGH must mimic all aspects of daily GH in terms of safety, efficacy and tolerability. These include increased bone growth and mineral deposition, muscle mass gain with improved exercise tolerance and enhanced lipolysis with body composition optimization, and yet, without increased immunogenicity, metabolic complications, injection site reactions, lipoatrophy or pain. Through decades of re-formulation and refinement, daily GH is available as a single, low volume, stable at room temperature injection administered via easy-to-use pens with fine gauge needles to minimize discomfort. These are administration characteristics that a LAGH must also satisfy.

The only LAGHs that have succeeded in providing both accelerated HV as well as correcting increased truncal adiposity – a measure of GH’s effect on metabolism – have been depot formulations, which release unmodified GH. Given GH receptor distribution throughout the body and the pleiotropic actions of GH, a viable LAGH would likely have to maintain the same organ and tissue distribution as native GH, e.g., a candidate based on unmodified GH.

## TransCon GH

### Background

Integrating the experiences of previous developments in the LAGH space, TransCon GH was developed as a sustained-release GH prodrug. It leverages the known pharmacology and distribution of unmodified GH in currently available, daily administered products with the properties of an inert PEG-containing carrier molecule. Recombinant human GH is transiently bound to methoxy polyethylene glycol (mPEG) via a proprietary TransCon linker. In contrast to permanent PEGylation, in which the PEGylated construct is the active entity, the TransCon carrier is optimized to inactivate the biological activity (i.e., receptor binding) of GH within the TransCon GH prodrug complex. This is done by applying a TransCon polymer carrier based on a four-arm structure where the branching points of the four arms are placed close to the protein surface. The mPEG acts as a carrier, inactivating GH, thereby creating a circulating GH prodrug with an extended circulation time in the body through reduced receptor-mediated elimination and renal excretion.

**Figure 1:**
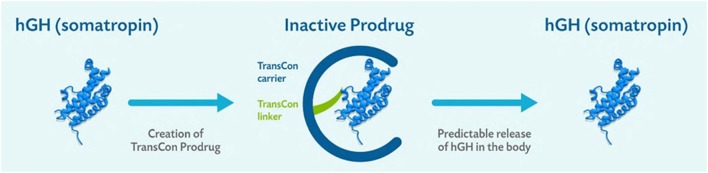
TransCon GH is a sustained-release inactive prodrug consisting of parent drug, unmodified GH, transiently bound to a carrier, mPEG (40 kDa), via a proprietary TransCon linker that is autohydrolyzed under physiologic pH and temperature. Reproduced under the terms of the original CCBY licence, from Chatelain P, Malievskiy O, Radziuk K, Senatorova G, Abdou MO, Vlachopapadopoulou E, Skorodok Y, Peterkova V, Leff JA, Beckert M, *et al.* A randomized phase 2 study of long-acting TransCon GH vs daily GH in childhood GH deficiency, *Journal of Clinical Endocrinology and Metabolism*, 2017, volume **102**, pages 1673–1682, (doi:10.1210/jc.2016-3776).

Based on physiologic pH and temperature, the TransCon linker autohydrolyzes following first-order kinetics, releasing fully active, unmodified GH over a one-week period designed to allow the same tissue distribution and receptor activation as endogenous GH (Fig. 1). The release of GH liberates the inactive carrier, allowing elimination from the body. PEG is cleared primarily by renal filtration and to a minor extent by hepatobilary excretion (68).

### Development

No adverse effects related to the mPEG carrier have been observed in extensive TransCon GH toxicology studies or clinical trials.

**Figure 2:**
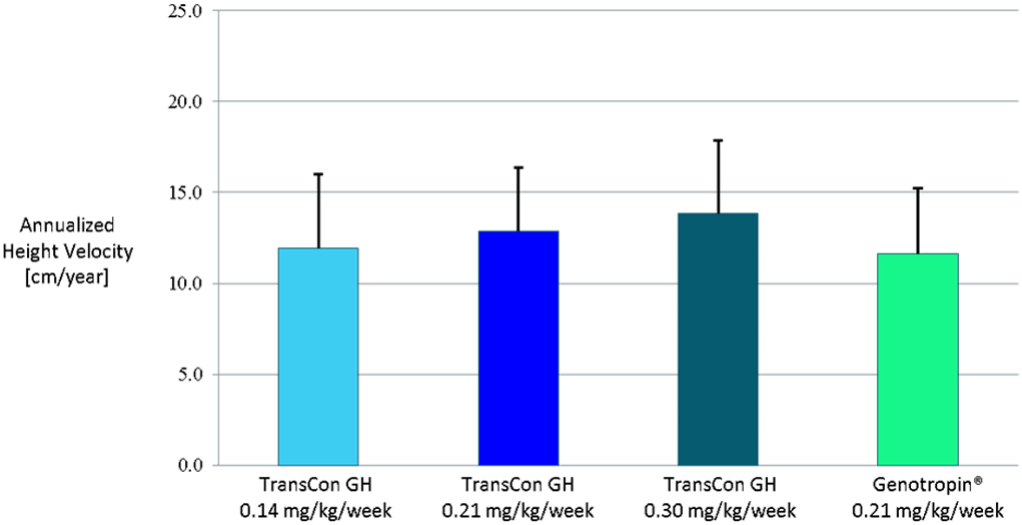
Annualized height velocity (mean + s.d.) in 53 subjects after 26 weeks of TransCon GH vs daily GH (Genotropin) treatment. Reproduced under the terms of the original CCBY licence, from Chatelain P, Malievskiy O, Radziuk K, Senatorova G, Abdou MO, Vlachopapadopoulou E, Skorodok Y, Peterkova V, Leff JA, Beckert M, *et al.* A randomized phase 2 study of long-acting TransCon GH vs daily GH in childhood GH deficiency, *Journal of Clinical Endocrinology and Metabolism*, 2017, volume **102**, pages 1673–1682, (doi:10.1210/jc.2016-3776).

In a phase 2 trial of children with GHD, TransCon GH (0.21 mg/kg/week) demonstrated that serum GH, as measured by the maximum GH concentration (*C*_max_) and area under the curve (AUC) over 7 days was within the physiological range and similar to a weekly cumulative dose of daily GH (Genotropin 0.21 mg/kg/week). IGF-1 changes demonstrated a dose–response relationship to TransCon GH while IGF-1 standard deviation score of all three TransCon GH doses normalized. Mean annualized HV ranged from 11.9 cm to 13.9 cm/year at a dose range of 0.14–0.30 mg GH/kg/week and compared favorably to 11.6 cm/year for 0.21 mg GH/kg/week of daily GH (Fig. 2) (69).

Adverse events were mild to moderate and most were unrelated to or unlikely to be related to the study drug. TransCon GH injection site reactions were similar to daily GH without lipoatrophy or nodule formation. One TransCon GH subject developed a low-titer, non-neutralizing antibody response; no neutralizing anti-GH binding antibodies were detected. Similar to daily GH, the mean body mass index SDS was stable across three TransCon GH cohorts (69).

In a phase 2 trial of adults with GHD, TransCon GH demonstrated a linear, dose-dependent increase in GH peak exposure without accumulation and a similar *C*_max_ as compared to daily GH (Omnitrope) at equivalent weekly dosing. IGF-1 exposure following equivalent dosing of TransCon GH and Omnitrope was also similar. Overall, TransCon GH was well tolerated. No lipoatrophy or nodule formation occurred at injection sites. No treatment-emergent anti-GH antibodies were detected (70).

Both the pediatric and adult GHD phase 2 results supported advancement of TransCon GH into phase 3 development.

## Discussion

TransCon GH was designed by integrating decades of learning to create a molecule with a once-weekly dosing profile that could match the safety and efficacy of daily GH. The objective was to provide more convenient drug administration to ease the lives of patients with GHD, thereby potentially improving compliance and treatment outcomes.

The Growth Hormone Research Society recently identified a number of potential safety issues relating to the development of LAGH (11). TransCon GH was designed to address these issues by predictably releasing unmodified GH, thereby maintaining the same mode of action and distribution as daily GH while optimizing PK. In phase 2 trials, TransCon GH produced peak GH levels similar to daily GH, and trough levels returned to baseline before subsequent dosing. In addition, as unmodified GH is released from the prodrug, similar efficacy is achieved on a molar basis compared to daily GH, enabling milligram to milligram conversion. Physiologic levels of GH, in turn, produced physiologic IGF-1 levels in a dose-proportional manner, with IGF-1 levels maintained within the normal range throughout the week (69).

TransCon GH was designed as a prodrug, with GH transiently bound to the inert mPEG carrier, which inacti­vates the GH molecule, preventing receptor-mediated binding and uptake by GH receptor-expressing tissues as well as renal clearance. With autohydrolysis of the TransCon linker, unmodified GH is gradually released, designed to maintain similar GH exposure levels and volume of distribution to those obtained with daily GH administration. Meanwhile, the mPEG carrier–linker complex, still bound together, is excreted separately.

While GHD patients generally do not develop neutralizing antibodies to daily GH, permanent GH modification creates a non-naturally occurring protein, increasing the risk of inducing anti-GH antibodies that may render GH therapy ineffective. Should this occur, patients may be in a situation similar to children with GH gene deletions who develop neutralizing antibodies to GH therapy and thus require daily IGF-1 administration to maintain growth. Since IGF-1 given alone lacks the direct GH effect on bone, it produces a lower annualized HV than daily GH and may increase adiposity due to unopposed IGF-1-mediated lipogenesis (4). In contrast, to date, TransCon GH has shown a lack of immunogenic potential similar to daily GH in phase 2 studies of both children and adults with GHD. Only one subject developed a low non-neutralizing anti-GH antibody titer that did not appear to affect PK or PD; the subject had an annualized HV of 19.0 cm/year (69).

While lipoatrophy in adults with GHD has been reported for several protein-enlarged LAGHs, GH activity of TransCon GH is masked upon SC injection. Upon absorption from the injection site, it acts as a circulating depot in the bloodstream, releasing unmodified GH over one week. Thus, local exposure at the injection site is minimized, reducing the risk of lipoatrophy. As expected, no lipoatrophy has been observed in children or adults with GHD to date.

Generally, 0.24 mg GH/kg/week of daily GH is used as a comparator in pediatric LAGH pivotal trials to reflect a globally accepted dose. However, to optimize outcomes, higher daily GH doses of approximately 0.30 mg/kg/week are commonly used in the United States. As such, a successful LAGH should demonstrate non-inferiority not only to the dose of daily GH used as an active comparator in pivotal trials but also those used in local practices. TransCon GH demonstrated an annualized 6-month HV that was similar to daily GH at doses commonly utilized in the United States, Europe and Japan (i.e., 0.14–0.30 mg GH/kg/week). This efficacy is by design; the small 22 kDa size of unmodified GH released from TransCon GH is, like daily GH, able to reach target tissue such as growth plates. Long-term safety and efficacy will be confirmed in an ongoing phase 3 trial in children with GHD.

## Conclusion

The ultimate goal of LAGH development is to optimize adherence to GH replacement therapy and thus improve patient outcomes in real world conditions. Except for administration frequency, all characteristics of daily GH must therefore be maintained, including safety, efficacy and tolerability, including lack of immunogenicity. A data review for both failed LAGHs and those currently in development reveals that a product delivering unmodified GH superimposed on an inert prolongation technology may be an appropriate design for a successful LAGH.

## Declaration of interest

K S is an employee of Ascendis Pharma A/S. E M, D K and J A L are employees of Ascendis Pharma, Inc.

## Funding

This work was supported by Ascendis Pharma A/S.
